# Accessibility of Park Green Space in Wuhan, China: Implications for Spatial Equity in the Post-COVID-19 Era

**DOI:** 10.3390/ijerph19095440

**Published:** 2022-04-29

**Authors:** Shujin Zhang, Peiheng Yu, Yiyun Chen, Ying Jing, Fanxin Zeng

**Affiliations:** 1 School of Resource and Environmental Science, Wuhan University, 129 Luoyu Road, Wuhan 430079, China; zhangshujin@whu.edu.cn; 2 Department of Building and Real Estate, The Hong Kong Polytechnic University, Hong Kong 999077, China; peiheng.yu@connect.polyu.hk; 3 Ningbo Institute of Technology, School of Economics, Zhejiang University, 1 Qianhu South Road, Ningbo 315000, China; y.crystal@nit.zju.edu.cn; 4 Department of Land Surveying and Geo-Informatics, The Hong Kong Polytechnic University, Hong Kong 999077, China; fanxin.zeng@connect.polyu.hk

**Keywords:** accessibility, two-step floating catchment area method, park green space, spatial equity, post-COVID-19 Era

## Abstract

During the COVID-19 pandemic, people have seen the precious value of park green space for health. In the post-COVID-19 Era, it is essential to understand the different needs and expectations of different communities for the use of park green space. A myriad of previous studies focused on the whole city’s demand for park green space, while few studies examined spatial equity from a supply-demand perspective. This paper aims to investigate the differences in park green space accessibility among people of different ages at a community scale. Specifically, to better evaluate the accessibility of park green space and account for the travel choice, we compared the effects of the two-step floating catchment area (2SFCA) method containing different distance decay functions (i.e., the improved 2SFCA methods) by considering the traffic network and the scale of park green space. In addition, we compared the improved 2SFCA methods with the traditional 2SFCA. This study investigated the spatial equity of park green space accessibility in 1184 communities with a total population of 6,468,612 in the central urban districts of Wuhan. The results showed that the high accessible communities were concentrated in the urban center along the Yangtze River. The improved 2SFCA methods outperformed the traditional 2SFCA, and presented smoother gradient information. It was revealed that over half of communities’ park green space accessibility levels did not match their population density. Inequality of accessibility to park green space was found in people of different ages, especially for the youth (Gini coefficient was as high as 0.83). The difference in the accessibility of urban park green space among different age structures implies the need to integrate community green space planning into urban planning in the post-COVID-19 Era.

## 1. Introduction

For more than half a century, most areas around the world have experienced rapid urbanisation. Urban populations accounted for 55% of the world’s population in 2018, and it was estimated that two-thirds of the population will live in cities by 2050 [1]. Explosive urban growth has profound implications for global health issues [2]. A high-density population leads to a high risk of urban disease transmission, making them incubators for infectious diseases, particularly the Coronavirus disease (COVID-19). COVID-19 as a highly infectious respiratory virus has now spread to over 200 countries and regions worldwide, becoming a major threat to global health [3]. The strict lockdown management implemented by the Chinese government in response to the COVID-19 pandemic makes houses the only place where people live, work and socialise. Recent research demonstrates that this lockdown may lead to serious psychological problems, such as emotional instability, depression and irritability [4]. People often feel anxious and depressed due to internet rumors, fear of illness and lack of exercise. Moreover, age and gender differences may affect the severity of the disease, and the elderly are one of the most vulnerable groups [5,6]. Simultaneously, public awareness of the importance of urban park green space has been strengthened by people’s desire to escape to the outdoors. Increasing urban green space has been shown to slow the spread of COVID-19 [7]. A survey of residents’ willingness to go to urban parks after lifting the lockdown in Wuhan on 8 April 2020, indicated that open spaces with vegetation were vital for recovery from lockdown-related mental health problems [8]. Venter et al. (2020) proved that leisure use of urban parks, surrounding forests and protected areas increased during the outbreak of COVID-19, which highlighted the significance of accessing park green space [9].

As a crucial component of modern cities, park green space provides the public with aesthetic enjoyment, entertainment, access to clean air and a relatively quiet space. These spaces can have environmental, social, health and economic benefits, such as improving air quality [10,11], promoting physical activities [12,13], mitigating the urban heat island effect [14], and enhancing social cohesion [15]. Regular exposure to park green space has positive implications for maintaining public physical and mental health [16]. For instance, spending more time in park green space has a positive effect on the treatment of mental diseases such as depression. In addition, an accessible park green space system is significant for achieving sustainable development goal 3 (good health and well-being) and goal 11 (resilient and sustainable cities) [17].

In geography, the concept of accessibility is generally defined in terms of spatial and non-spatial dimensions. From the perspective of spatial accessibility, the concept was first defined by Hansen as the chance of interaction between points in traffic network space [18]. Spatial accessibility is also referred to as ‘explicit accessibility’ and includes availability and accessibility, which reflects the spatial impediments and time costs of travelling from a starting point to an area [19,20]. It denotes the efficiency of spatial organisation [21]. From the non-spatial accessibility perspective, it includes accommodation, affordability and acceptability. Non-spatial accessibility focuses on the influence of non-geographic elements, which reflects the subjective willingness of individuals to travel to certain types of facilities, the attractiveness of the facilities themselves, and the preferences of users [22,23]. Potential geographic accessibility refers to the future availability of facilities that is possible, potential, but not actually occurring, such as measuring the relationship between supply and demand using a two-step floating catchment area method. It is used to assess the nature and pattern over space of physical access to service facilities and can be defined as simply “the presence of enabling resources”. In conclusion, accessibility involves the relationship between humans and space and features the concept of time. This paper focuses on the potential geographic accessibility of park green space and its spatial variation.

Various accessibility evaluation methods have been developed over the last few decades. The commonly used methods could be summarised into three categories: (i) the proportion method, to count the green spaces in the area and estimate accessibility [24]; (ii) the distance method, which uses Euclidean distance or network distance to the nearest green space to measure accessibility [25,26]; and (iii) the gravity model, which introduces the law of gravitation potential energy, and calculates the sum of energy exerted by all green spaces by combining the attractiveness of park green space and residents’ demands [27]. The former two methods do not consider the landscape heterogeneity and the user’s subjective choice. They assume that people will choose the nearest green space. However, in fact, people have more choices. Although the basic form of the third method considers the possibility of multiple choices for people, it does not consider the relationship between the supply and demand for green space and the population. In response to these limitations, Radke and Mu (2000) first proposed the two-step floating catchment area (2SFCA) method to measure accessibility. Based on the basic form of the 2SFCA method [28], various improvements have been made in terms of boundary [29,30], distance decay [31,32] and supply and demand [33]. These improvements make the 2SFCA method more accurate and comprehensive, enhancing its applicability for different scenarios. The scale of park green space and traffic network largely influence the travel choice of people. For example, the facilities of parks determine the services that can be provided, which implies that a large park, with adequate facilities, has a bigger radiation radius compared to a small park. At the same time, the convenience of travel generally has an impact on travel. However, these two factors are rarely considered in the traditional modeling of 2SFCA. Moreover, few researchers have compared the results of different distance decay functions.

The research on accessibility has mostly focused on access to public services. Public services are resources directly or indirectly provided by the government for the public and shared by all. In general, studies on public service accessibility have experienced three stages: regional equity, spatial equity and social equity. In the 1960s and 1970s, relying on the background of the welfare society, equity was reflected in the equal access to resources and services for all people. Uniform distribution was the central concept in this period, but the actual needs of people and the spatial layout of facilities were not considered [34]. After the 1970s, equity researchers began to pay attention to the number and layout of facilities. GIS techniques were used to calculate accessibility indexes to reflect the distance or cost of getting people to their destinations [35]. At the beginning of the 21st century, economic globalisation and cultural diversity put forward new demands for equity. It requires governments to take into account the different needs between social groups to provide public services. The accessibility evaluation method also started from the two aspects of facility supply and user to make the results more targeted [36]. At this stage, the research generally involved difference description, equity evaluation and mechanism explanation. Among them, difference description includes the attributes of users and service facilities, accessibility relationships, characteristics and patterns of use. Equity evaluation includes evaluation principles, methods and characteristics analysis. Mechanism explanation refers to the analysis of factors and formation mechanisms that affect inequity through the interaction between users and public services. Previous studies have suggested that age, gender, ethnic characteristics and social class affect access to park green space, in which economic status and age play a more important role [37,38]. Recent studies have found that vulnerable groups (the elderly, low income, black people, etc.) are more likely to lack access to high-quality green space during the pandemics, reflecting the existence of social inequity [39]. With the promotion of the people-oriented concept in planning and design, the spatial disparity between the provisions of park green space and the needs of residents has become increasingly concerned in developed and developing countries [40,41]. Nevertheless, previous studies have mainly focused on the demand for park green space, and little attention has been devoted to whether the resources of each district match the needs of residents.

Minimum accessibility to park green space may be necessary in order to meet the basic needs of residents. Understanding variation in accessibility can also reveal the spatial dimension of concerns about inequality [42]. We hereby compare the improved 2SFCA methods to evaluate the spatial equity of park green space accessibility among people with different age structures in Wuhan. The specific objectives are (i) to improve the delineation of park green space accessibility by using three improved methods that consider the scale of park green space and traffic network; (ii) to investigate the spatial relationship between population density, housing price and park green space accessibility; and (iii) to reveal the spatial inequity of park green space services among different age structures. Our study can generate more generalised knowledge that provides a framework for resilient, livable and healthy cities.

## 2. Data and Methods

### 2.1. Study Area

**Figure 1:**
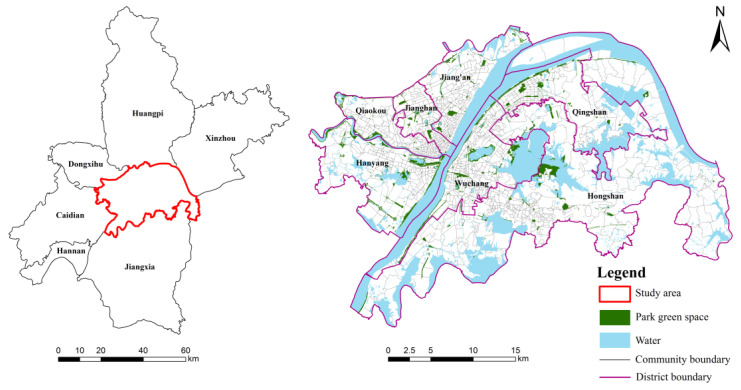
The study area and spatial distribution of park green space in Wuhan.

Wuhan is the largest city in central China and the capital of Hubei province, located at the intersection of the Han River and the Yangtze River. By the end of 2019, the city had 13 districts with a total area of 8569.15 km^2^, a registered population of 9.08 million and a floating population of 5.10 million. Wuhan has issued a series of detailed policies on increasing urban green space for each administrative region since 2001. These policies have greatly improved the environment and quality of life. By the end of 2019, Wuhan city’s green space area reached 258.66 km^2^. The green space rate was 35.68%, and the park green area per capita was 9.61 m^2^. Our study area is in the central urban district, which includes Jianghan, Hanyang, Qiaokou, Jiang’an, Wuchang, Hongshan and Qingshan, covering an area of 863 km^2^ (Figure 1). The population in the central urban district accounted for approximately 60.28% of the total population in 2019.

**Figure 2:**
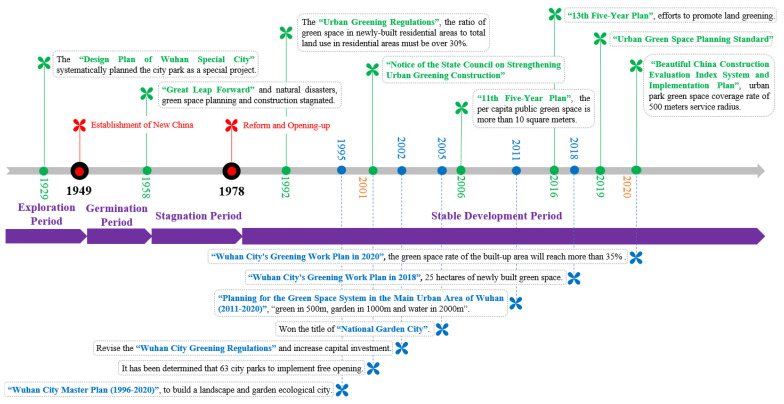
The development of park green space policy in China includes four stages: 1929–1949 exploration period, 1949–1958 germination period, 1958–1978 stagnation period and 1978—present stable development period.

Local governments have dedicated time and efforts to green benefits. Before the founding of the People’s Republic of China, Wuhan did not form a complete urban green space system. After the implementation of the Design Plan of Wuhan Special City in 1929, urban parks began to be planned systematically as a special project. This period was called the exploration period of the park green space policy (Figure 2). In the 1950s, Wuhan City formulated a three-town-integrated urban master plan, which started the germination period of urban green space system planning and construction. Subsequently, due to the rise and fall of politics and natural disasters, the construction of green space in Wuhan experienced a period of stagnation from 1958 to 1978. A large number of green spaces were occupied, demolished and destroyed. After the reform and opening-up, urban construction was on the ascendant all over the country, and the urban green spaces in Wuhan have now entered a period of stable and rapid development. During this period, urban greening mainly emphasised the quota index such as green space ratio, green space coverage rate and per capita public green area. The sustainable development of cities needed to evolve towards an ecological pattern. In 1995, the Wuhan City Master Plan (1996–2020) proposed to build Wuhan into an ecological city with landscapes and gardens rich in waterfront characteristics. In 2005, Wuhan was awarded the title of “National Garden City”. Based on the principle of people-oriented and fair sharing, in order to promote the spatial equity in park green space, the Wuhan Natural Resources and Planning Bureau compiled the Planning for the Green Space System in the Main Urban Area of Wuhan (2011–2020) combined with the distribution of existing landscape resources in 2011. The goal is to achieve green space of 16.8 square meters per capita in urban parks in the main urban area through a rational layout of the green space system. It also aims to build green landscape with “500 m of greenery, 1000 m of gardens, and 2000 m of water”, making Wuhan a “green city of rivers” with a good living environment.

### 2.2. Data Sources

Multi-source data were integrated and utilised in our study: (i) The spatial distribution and area of park green spaces were derived from the urban land status survey and the Green Space System Planning Reports of Urban Center in Wuhan from 2011 to 2020. According to the concept of park green space and previous literature, we selected 748 park green spaces in the study area, mainly including various parks (such as comprehensive parks, zoos, botanical gardens, etc.) and roadside green spaces (green areas with recreational function, including greenway and green square). (ii) The road network data and the built-up area data were provided by the Wuhan Natural Resources and Planning Bureau (http://zrzyhgh.wuhan.gov.cn/, accessed on 19 December 2019). (iii) The community and population data were obtained from the census data of Wuhan in 2016. All spatial data were registered using a uniform spatial coordinate system to ensure data compatibility.

### 2.3. Accessibility of Park Green Space

In order to understand the spatial presentation of accessibility calculated by different distance decay functions, to help us recognize nuances and better assess spatial inequity, we used four methods to measure the accessibility of park green space, including the traditional 2SFCA method (2SFCA) [43], the road-based kernel density 2SFCA method (RKD2SFCA) [44], the road-based Gaussian 2SFCA method (RG2SFCA) [45] and the road-based gravity 2SFCA method (RA2SFCA) [46]. In addition, we divided park green space into three levels based on the actual area of parks in Wuhan and the Standard for Planning of Urban Green Space (GB/T 51346-2019) in China. Accessibility is also estimated according to different levels and service radius of the park green space: (i) City-level park provides places for rest, exercise and various collective cultural activities (>0.2 km^2^); (ii) District-level park refers to the centralised green space with certain activities and facilities, which serves the residents within a certain range of residential land (0.05–0.2 km^2^); (iii) Neighborhood-level park is a small-scale green space designed to provide daily entertainment for residents (<0.05 km^2^). As the service radius of park green space is mainly related to its scale and function, referring to the radius set in the Standard for Planning of Urban Green Space (GB/T 51346-2019), the service radii of the three types of parks defined above are designated as 3000 m, 1500 m and 500 m, respectively. The network analyst tool in ArcGIS is used to calculate the shortest walking path from each residential area to the park through the traffic network instead of Euclidean distance. Since the origin point and the destination point are not both on the road network, an auxiliary line from the origin point or the destination point to the road is constructed, and the shortest distance from the point to the road is adopted. In this paper, the centroid of the park and community is taken as the origin point or destination point.

The traditional 2SFCA method is based on supply and demand respectively, and it is divided into two steps to calculate the accessibility of park green space. The specific calculation is as follows:

In the first step, for each green space j, given a spatial distance threshold *d*_0_, a spatial catchment is formed. Search for the population of all communities *k* from a given location *j* to the threshold distance ($d_{0}$). Then all communities *k* within the threshold distance ($d_{0}$) from the given location *j* are searched. The ratio ($R_{j}$) of the supply to the demand is expressed as:

(1) $$R_{j}=\frac{S_{j}}{\sum_{k\in \left\{d_{kj}\leq d_{0}\right\}}G\left(d_{kj},d_{0}\right)P_{k}}$$

In Equation (1), $P_{k}$ is the population at location *k* within the catchment ($d_{kj}\leq d_{0}$) of green space *j*. $d_{kj}$ is the spatial distance from location k to location j. $S_{j}$ is the area of green space at *j*. $G\left(d_{kj},d_{0}\right)$ is the distance decay function used in the next several methods, and here is coefficient 1.

In the second step, for each community i, given the spatial distance threshold $d_{0}$, a spatial catchment is formed. Similarly, all the park green spaces within the service threshold ($d_{0}$) are searched and the supply ratio $R_{l}$ to obtain the green space accessibility *A_i_* of community i can be calculated using Equation (2).

(2) $$A_{i}=\sum_{l\in \left\{d_{il}\leq d_{0}\right\}}G\left(d_{il},d_{0}\right)R_{l}$$

In Equation (2), $R_{l}$ represents the supply ratio of green space l in the catchment ($d_{il}\leq d_{0}$) of community i. All other notations are the same as in Equation (1). The centroid of the park and community is taken as the origin point or destination point.

The RKD2SFCA method uses Equation (3) as the distance decay function. It has been shown that the Epanechnikov function performs the best among different kernel functions and it produces the smallest mean integrated squared error (MISE) [47,48]. The letter represents the same meaning as above.

(3) $$G\left(d_{kj},d_{0}\right)=\frac{3}{4}\left[1-{\left(\frac{d_{kj}}{d_{0}}\right)}^{2}\right],\;d_{kj}\leq d_{0}$$

The RG2SFCA method uses Equation (4) as the distance decay function. The steps and meaning of letters are the same as above.

(4) $$G\left(d_{kj},d_{0}\right)=\frac{e^{-\left(\frac{1}{2}\right)\times {\left(\frac{d_{kj}}{d_{0}}\right)}^{2}}-e^{-\left(\frac{1}{2}\right)}}{1-e^{-\left(\frac{1}{2}\right)}},\;d_{kj}\leq d_{0}$$

The RA2SFCA method uses a power function as the distance decay function. The steps and meaning of letters are the same as above.

(5) $$G\left(d_{kj},d_{0}\right)=d_{kj}{}^{-\beta },\;d_{kj}\leq d_{0}$$

where β is the distance decay parameter. According to the previous studies [49,50], the value of β is between 1 and 2. When residents adopt low-cost transportation models such as walking, the distance decay parameter of 1.5 conforms to the suppressing effect on the speed of transportation models [51]. Therefore, 1.5 is chosen as the distance decay parameter.

### 2.4. Lorenz Curve and Gini Coefficient (GC)

The original intention of the Lorenz curve was to explore the distributional equity of the national economy, that is, the curve is formed by the cumulative income of the corresponding percentage of the population sequence with increasing levels of wealth [52]. On this basis, the Gini coefficient is proposed to quantify the concept of income inequality to evaluate the income gap in a certain area [53]. The Lorenz Curve can be used to measure environmental equity for different age groups by analyzing the distribution of park green space resources for different populations. According to the age standards of the Law of the People’s Republic of China on the Protection of Minors and the Law on the Protection of Rights and Interests of the elderly, youth (under 18 years old), young and middle-aged people (18–60 years old) and the elderly (over 60 years old) are divided. The horizontal axis is the percentage of the population, and the vertical axis is the cumulative percentage of the accessibility of the park green space.

The GC is a global evaluation index to evaluate the social equity of the allocation of park green space. The calculation formula is:

(6) $$G=\overset{n}{\underset{i=1}{\sum }}X_{i}Y_{i}+2\overset{n}{\underset{i=1}{\sum }}X_{i}\left(1-V_{i}\right)-1$$

In Equation (6), *n* is the number of communities. $X_{i}$ is the ratio of the population of the *i*th community to the total population. $Y_{i}$ represents the ratio of the park green space accessibility value of the *i*th community to the total value. $V_{i}$ is the cumulative value of the *i*th community’s park green space accessibility sorted from small to large. The Gini coefficient ranges from 0–1. The closer the coefficient is to 0, the more equitable the distribution of park green space among the population is, and the closer it is to 1, the more concentrated the distribution of park green space among the population is.

## 3. Results

### 3.1. The Pattern of Park Green Space Accessibility

Park green spaces are concentrated in the urban development core area, the areas with rich natural resources, both sides of the Yangtze River and the traffic lines, which are scattered and unevenly distributed as a whole (Figure 1). The grade and scale of park green space vary considerably from district to district. The park green space mainly diverges around the large-area park as the core, distributed in a radial pattern of the network. However, the connection between the park green space is relatively lacking, and there is almost no park green space in the east of the main urban area, which harms the function of the urban park green space and urban green space system.

**Figure 3:**
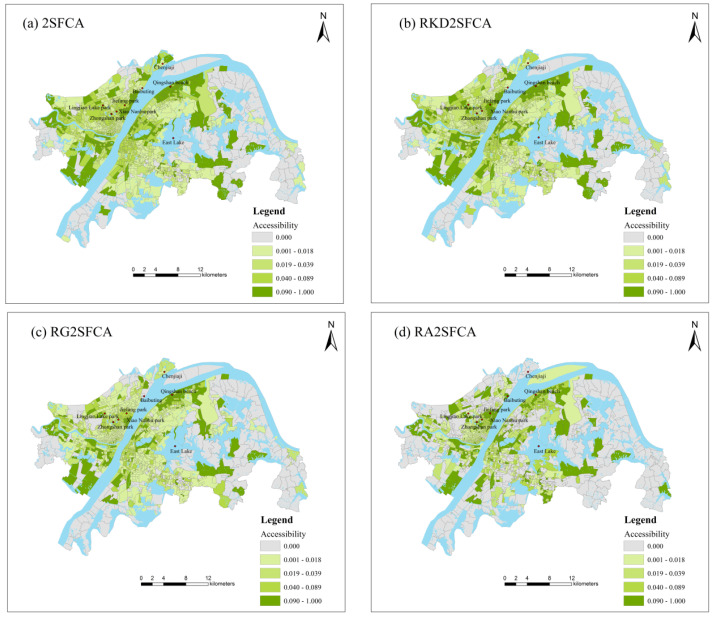
Accessibility of park green space in Wuhan under four methods: (**a**) the two–step floating catchment area method (2SFCA), (**b**) the road–based kernel density 2SFCA method (RKD2SFCA), (**c**) the road–based Gaussian 2SFCA method (RG2SFCA) and (**d**) the road–based gravity 2SFCA method (RA2SFCA).

As shown in Figure 3, the accessibility of 1184 communities in the central urban districts of Wuhan are analysed based on the 2SFCA and its improved form. The park green space accessibility of community is divided into five levels by the Natural Breaks (Jenks), including lower, low, medium, high and higher. The spatial patterns of park green space accessibility calculated by the four methods are similar. RG2SFCA and RA2SFCA show a more notable gradient in the accessibility of park green space. Taking the northern part of Qingshan District as an example, the 2SFCA method reveals that there is high park green space accessibility here. In contrast, the RA2SFCA method suggests that it is mixed with medium and low park green space accessibility, which may be related to the smoothing effect of distance decay function in the supply–demand interaction.

From the study area scale, the distribution of park green space accessibility presents significant spatial differences, and the high value of accessibility is manifested along the axis of the Yangtze River and Han River. It demonstrates that the Yangtze River Beach in Wuhan has a certain effect on the park green space accessibility. Among them, the higher accessibility is concentrated in the city center along the Yangtze River, while the areas with relatively lower accessibility are distributed on the edge of the city, especially in the eastern and southern regions.

From the community scale, the lower accessibility regions centralize in the areas with high population density, as well as the zones distributed in the fringe of central urban districts. The dense population region is mainly distributed in the old urban area, with insufficient park green space. Hence, the ratio of green space per capita is low and the accessibility is low. As for the urban−rural fringe zones, including Chenjiaji, Baibuting, and some communities along the third ring road, they are still in a developing stage with a lagging park green space construction. Therefore, they all present low accessibility to park green space. On the contrary, the high accessibility regions own the features of accessing group parks, nearing large-scale parks, or adjoining the specific beach. The group-park-region, such as places around Zhongshan Park, Jiefang Park, Xiao Nanhu Park and Lingjiao Lake Park have high accessibility. One reason is that the surrounding residents usually have multiple parks to choose from, hence can enjoy sufficient park green space service. Another zone is communities close to large-scale parks. These parks, mainly situated in the East Lake Scenic Area of Wuhan, occupy abundant green spaces and can satisfy citizens’ leisure requirements and other comprehensive functions, e.g., ecological protection. Beneficial to the diverse green space resources in the scenic area, the accessibility of this area is also high. The most specific case is the Qingshan Bin Jiang Business District. It has the highest green space proportion, about 80%, among the beaches in Wuhan. It has adequate park green space resources (six green parks and five urban greenways), making this area a highly accessible area.

### 3.2. Comparison of Different Methods

For a better comparison of the four methods, we calculate the average accessibility of park green space in each administrative region using a population-weighted mean (Table 1). In terms of administrative scale, the average accessibility value of park green space in Qingshan District is the highest in the 2SFCA method, followed by Hanyang District. Among the RKD2SFCA, RG2SFCA and RA2SFCA methods, the average accessibility value of park green space in Hanyang District is the highest, followed by Qingshan District. It can be seen that the accessibilities of Qingshan District and Hanyang District are better than that of other administrative regions because the number of underserved communities is less than that of other districts. Among the four methods, Qiaokou District and Jianghan District have the lowest average accessibility values, as they have less park green space distribution. Regardless of the accessibility measurement, the spatial distribution of underserved communities is clustered throughout the city.

**Figure 4:**
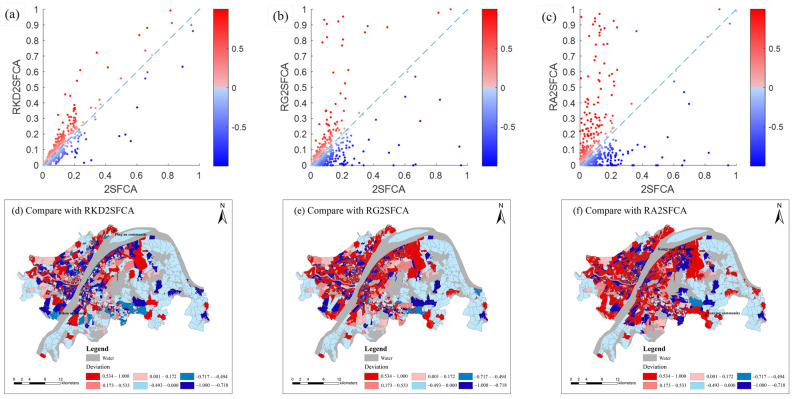
Comparison of 2SFCA method with RKD2SFCA (**a**), RG2SFCA (**b**) and RA2SFCA (**c**). The first row is the comparison of over/underestimation among methods. The second row is the over/underestimated spatial distribution map of 2SFCA method compared with RKD2SFCA (**d**), RG2SFCA (**e**) and RA2SFCA (**f**).

Different accessibility methods are further compared graphically using a scatter plot in Figure 4a–c with the 1:1 reference line, along which the traditional 2SFCA index is equal to the RKD2SFCA, RG2SFCA and RA2SFCA index. The points falling above or below the line indicate that the traditional 2SFCA method is higher or lower than the spatial accessibility obtained by the RKD2SFCA, RG2SFCA and RA2SFCA methods. Figure 4d–f shows the areas where the accessibility values calculated by the tranditional 2SFCA are lower (represented by negative values), equal and higher (represented by positive values) than those obtained by other methods. Figure 4a highlights that compared with RKD2SFCA, the park green space accessibility obtained by the 2SFCA method has a general correspondence among communities. However, there is also a phenomenon that relatively higher values are generated in areas with poor accessibility, and lower values are generated in areas with good accessibility. For example, the accessibility of Ping’an community in Jiang’an District is 0.053 in the RKD2SFCA method, while the value is 0.100 in the 2SFCA method. In the RKD2SFCA method, the accessibility of Dihou community in Wuchang District is 0.132, while the value in the 2SFCA method is 0.069. Figure 4b shows that the 2SFCA method generally overestimates the park green space accessibility for the RG2SFCA method, with a small number of values above the 1:1 reference line. The RG2SFCA method presents larger areas of low accessibility than the 2SFCA method (see the middle of Jianghan District and the southern areas of Jiang’an District in Figure 4e). In other words, the RG2SFCA method can identify local pockets of poor access in cities. Figure 4c indicates that compared with RA2SFCA, the 2SFCA method also systematically overestimates. The 2SFCA method produces some higher values in regions with lower accessibility, such as Sanxing community in the south of Hongshan District and Kangyuan community near the Qingshan Beach. This might be related to the smoothing effect without distance decay of supply–demand interactions.

### 3.3. Spatial Equity of Park Green Space Distribution

**Figure 5:**
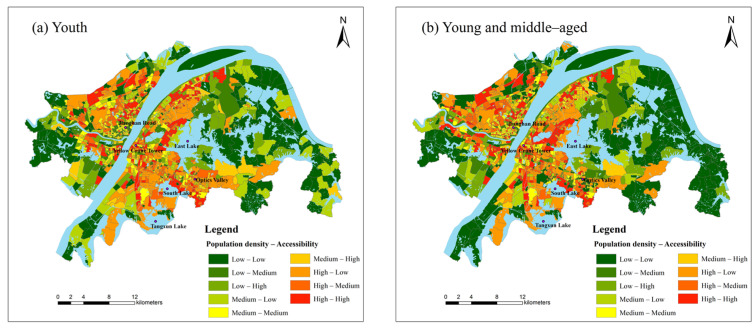

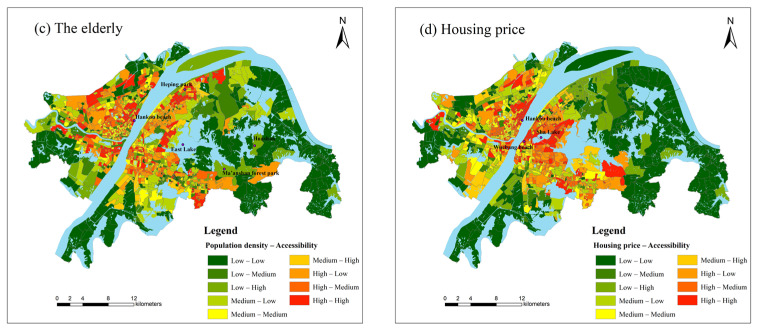
The matching of population density, housing price and park green space accessibility: (**a**) youth, (**b**) young and middle–aged, (**c**) the elderly and (**d**) housing price.

**Figure 6:**
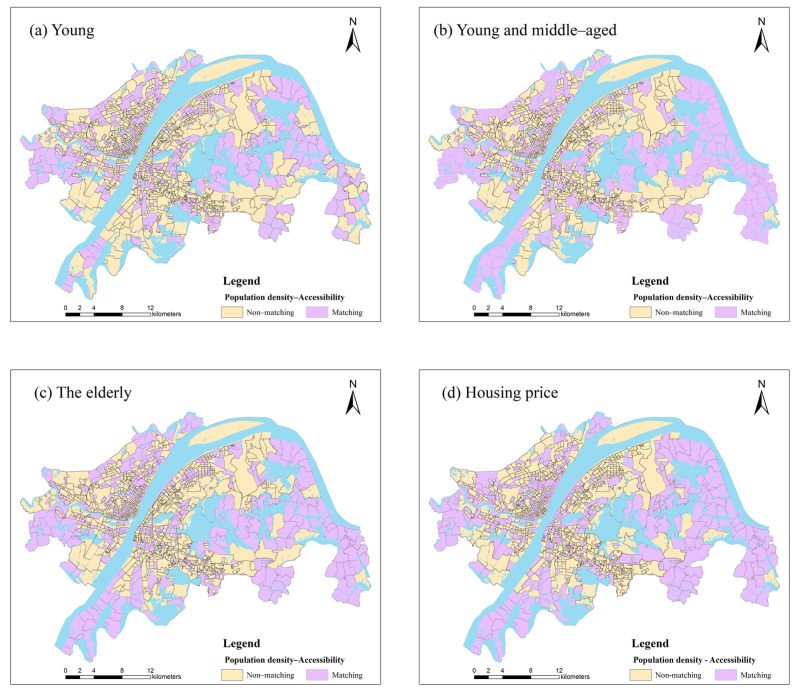
Spatial distribution of matching and non–matching between population density, housing price and park green space accessibility: (**a**) youth, (**b**) young and middle–aged, (**c**) the elderly and (**d**) housing price.

Residents’ demand for park green space will decrease with the increase of distance [54]. This supply–demand relationship shows a normal distribution with the change of distance, which is consistent with the performance of the Gaussian function [55]. Therefore, the RG2SFCA method is chosen to conduct the following research in this paper. We use the Natural Breaks (Jenks) to divide the accessibility, population density and housing prices into three levels: low, medium and high. Then the population density, housing prices and accessibility areas are overlapped respectively (Figure 5). For more visual display, we take the low-low, medium-medium and high-high levels as “matching”, and the rest as “non–matching” (Figure 6). As shown in Figure 5, the accessibility of park green space for youth and young and middle-aged people has similar distribution characteristics in a circle, with low accessibility in the outer circle. The matching degree of supply and demand in each circle layer is heterogeneous. The population density of the two groups matches the accessibility in the Jianghan Road Commercial District, the Yellow Crane Tower Scenic Area, and the north side community of Tangxun Lake. In the northwest of South Lake and East Lake, the Optics Valley Commercial District has a low degree of matching, which shows characteristics of “high population density–low accessibility”. There are more communities with dislocation of supply and demand, which means that the efficiency of park green space is inequitable. The region gathers colleges including Wuhan University of Technology and Wuhan University of Science and Technology, with a large number of young and middle–aged teachers and students, and is densely populated. As an important transportation hub and commercial center in Wuhan, the Optics Valley Commercial District is surrounded by many well-known universities and scientific research resources. The age structure tends to be young and middle–aged and the supporting park green space resources are few. The population density of the elderly in the Hankou Beach, Heping Park group and the west side of the East Lake have a higher matching degree with accessibility. Areas with low population density and high accessibility are occasionally distributed in the southeast of East Lake, Huashan Avenue and the south of Hanyang District. These areas have resources such as Ma’anshan Forest Park and Huashan Ecological Green City.

From a statistical point of view (Table 2), the relationship between park green space accessibility and population density is not notable compared with the change of community number in youth and young and middle-aged people. The matched population only accounted for 24.8% and 24.18% of the corresponding population respectively. Approximately 12% of the communities have high population density and high accessibility of park green space, while less than 18% of the communities have low population density and low accessibility of park green space. This suggests that over half of communities’ park green space accessibility does not match their population density. Among the elderly, communities with low population density and high accessibility account for 7.69%, while those with high population density and low accessibility account for 8.78%. The populations of these communities account for 19% of the total elderly population, and the imbalance between supply and demand in these communities should be optimized in time.

There is a close relationship between the environment and house prices, which is also the focus of continuous attention for governments and residents at all levels [56,57]. As can be seen from Figure 5, the match between housing price and accessibility in Wuhan is roughly distributed in circles to the periphery of the city. The greening rate has a promoting effect on the increase in housing prices. Communities with high housing prices and high accessibility are concentrated in the Hankou Beach, Wuchang Beach and the surrounding communities of Sha Lake. They account for 12.08% of all communities and have 13.24% of the total population (Table 3). Most of the communities with low housing prices and low accessibility are located outside the central urban districts, accounting for 18.92%, such as the southwest and northeast of Hongshan District and the northwest of Hannan District. The population density here is relatively low, the demand for housing is not too intense, but the park green space resources are also lesser.

**Figure 7:**
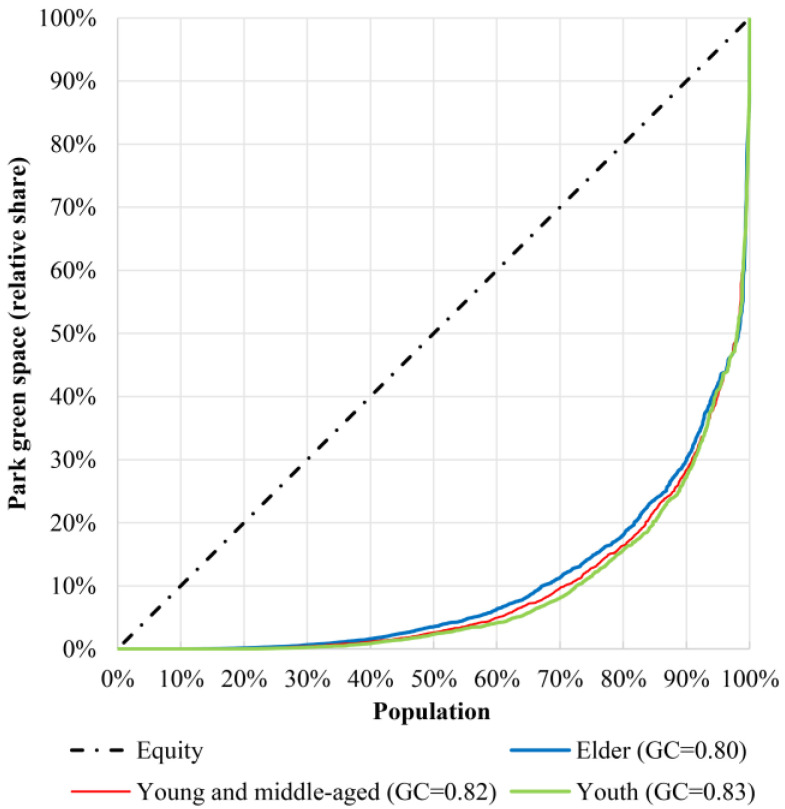
Lorenz curve and Gini coefficient of different age structure and park green space accessibility.

The Lorenz curve and Gini coefficient are introduced into the evaluation of the equity of park green space allocation and the results are shown in Figure 7. There are certain differences in the configuration of park green space among different age groups. Among the elderly, 80% of the community population shares only about 18% of the park green space services, in contrast, 20% of the population shares 82% of the park green space. The situation of youth and young and middle-aged people sharing park green space is the same or even worse. The Gini coefficient between the accessibility of park green space and the distribution of youth in the study area is 0.83, which is slightly larger than that of young and middle–aged and the elderly (GC = 0.82 and 0.80), indicating that the inequality of the park green space service for youth is particularly remarkable. On the whole, the resource allocation of park green space in the central urban districts of Wuhan is relatively unbalanced, and there is a certain spatial mismatch with the distribution of population resources, which is contrary to the concept of “space sharing” of green spaces.

## 4. Discussion

### 4.1. Park Green Space, Spatial Equity and Urban Planning

The urban green space network system is the foundation of urban ecological security and the green barrier to prevent the spread of viruses [58]. The lockdown caused by the COVID-19 pandemic once again strongly demonstrated the importance of park green space for people’s health and well-being. The results indicate that the three improved 2SFCA methods are superior to the traditional 2SFCA method, and can display more abundant gradient information of park green space accessibility. The traditional 2SFCA method assumes that the distance decay in the catchment is negligible, which means that individuals may also get services from nearby and catchment boundaries. This is not the case in large cities with a widely dispersed population. Different methods reach an agreement about low park green space access areas, which reflects the actual use degree of park green space. Low accessibility mainly occurs in the east and southwest of Hongshan District, the west of Hanyang District and the north of Jiang’an District. The improved 2SFCA methods are intuitively preferable to the traditional 2SFCA, but it is difficult to define an appropriate distance decay function that conforms to the real behavior of the population. Investigating more regions can better understand the differences between these methods. Urban park green space does exhibit spatial inequality among people with different age structures, which is consistent with many research results [59,60]. Communities with high housing prices, as well as those with a higher proportion of the elderly, usually have better opportunities to park green space. These results reflect the trend in the literature in most countries that vulnerable groups tend to be disadvantaged in terms of access to public resources [61,62]. This proves the necessity for popularising park green space in the Sustainable Development Goals.

From the perspective of spatial equity theory, the different class status of urban residents brings about different spatial rights and interests, which makes it difficult for vulnerable groups to enjoy equal access to social security and public resources [63]. In the post-COVID-19 Era, the crisis has provided us with some opportunities to prompt more people to reflect on the coexistence model of humanity and nature. It is not just about restarting, but about reimagining, designing and investing in new public spaces. One of the functions of urban planning as a key instrument of government intervention in urban development is the spatial distribution of public facilities services, so spatial equity is necessarily an allocation criterion to be considered [64,65]. Without the guidance of spatial equity, an increase in public service facilities would not necessarily improve the whole population to equally share resources, but may lead to a more polarized situation. As a result, conflicts of interest between different social groups occur, which affect social harmony and stability.

On the urban scale, the reasonable distribution of park green space can effectively disperse architectural space, reduce population density, and help prevent the spread of the epidemic. Park green space has the characteristics of open space, sufficient light and good ventilation, which can dilute the possible presence of viruses in the air and greatly reduce the infection rate [66]. For urban managers and planners, planning is a process of removing the interference between realistic conditions and future goals. The bottom-up thinking model based on the problem is of equal importance to the top-down thinking model starting from the goal [67]. Urban development can increasingly adopt the principle of ‘smart growth’ [68,69], paying attention to addressing the distorted allocation of scarce space resources and giving full play to the overall guiding role of urban planning and the decisive role of the market in allocating resources. In China, the ratio of per capita park green space is a commonly used indicator in urban park planning [70,71]. The development of urban parks often emphasises scale and quantity, while less attention is paid to the matching relationship between the spatial distribution of urban parks and the needs of residents. Meanwhile, the city is required to be people-oriented, fully embody the inclusiveness of society, and focus on the material and spiritual needs of all people in the city [72]. At the same time, it is important to pay attention to the change in urban population structure, grasp the demand characteristics and development trends of people more accurately, and provide differentiated planning support according to the spatial needs of different types of people. For example, youth may prefer sports-based activities such as football and tennis [73]. The elderly prefer to walk quietly or chat and play chess in parks with flat roads [74,75].

**Figure 8:**
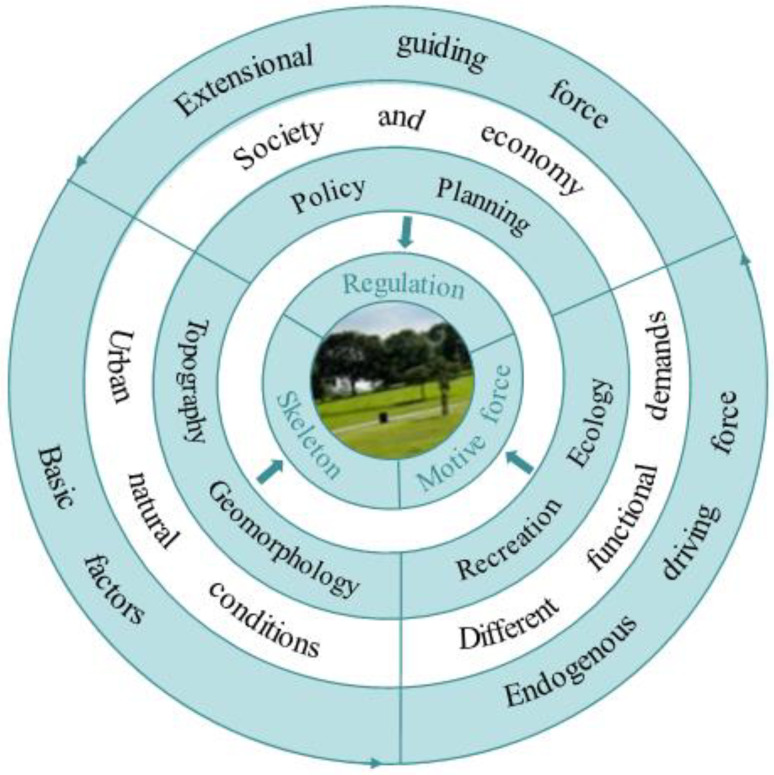
The development mechanism and planning framework of park green space.

The layout structure and network system of urban park green space could be improved to tackle the challenge in the post-COVID-19 Era. It is better to minimize the service blind areas of park green space and construct the urban park green space system with complete structure and complementary functions (Figure 8). The south of Hongshan District and Jianghan District, the east of Wuchang District and the central of Qiaokou District are the weak areas of the urban park green space system in Wuhan city. In old urban areas (such as Qiaokou District and Jianghan District), the density of residential buildings is relatively high, and the available land resources are limited. The urban micro-space and unused space should be fully utilized to increase small pocket parks and street green spaces [76]. In the periphery of the central city, there are more land resources available for development, and the natural resource background is superior. Based on the good landscape resources combined with the distribution of residents, some high-quality large-scale park green spaces can be designed and developed, so as to increase residents’ access to park green space. In addition, it is also significant to improve the service capacity and environmental quality of the existing park green space, especially to renovate and upgrade the park green space that is too small to provide support for residents’ recreational activities. In areas with relatively abundant land and construction conditions, it is easy to expand the area of park green space, increase public toilets, fitness and leisure supporting facilities, and improve the supply level and attractiveness of the facilities. In future research, residents’ satisfaction and demands for park green space need to be strengthened [77,78], and the effective expression mechanism of residents’ needs should be paid attention to. With the development of big data technology, thinking about how to characterize the social-economic status of residents through objective data, and taking it as a vital indicator to measure equity, are necessary goals, which have certain enlightening significance for the construction of user demand analysis [79].

### 4.2. Limitations and Future Research

Several limitations should be mentioned in relation to this study. First, due to data limitations, there is a certain time difference between the obtained data, and we evaluated the accessibility of park green space in the walking model without considering accessibility in multiple traffic models, which may have an impact on the results. Future research could consider other transportation models, especially the impact of urban public transport systems on accessibility. Since accessibility is a characteristic attribute of multiple locations, it is highly dependent on the available transportation modes. Second, although the level and service radius of park green space was considered, it did not involve the characteristics and preferences of specific users, as well as the entrance and infrastructure of the park green space. Further studies are thus needed to expand and enrich the methods and findings of these aspects. Finally, the Gini coefficient and Lorenz curve can only estimate the inequality within groups, and new instruments and methods are needed to explore the inequality between groups. The influence of these factors should be considered further in the future. It is possible to analyse how the level of access to park green space in each community compares to the targets set in the latest city plans.

## 5. Conclusions

The COVID-19 pandemic has elevated the values of park green space and has underscored the benefits for cities in creating more park green space and more equitable access to them. Taking Wuhan as an example, three improved 2SFCA methods were used to assess accessibility by considering the traffic network and the scale of park green space. Whether the distribution of park green space is equal to different age groups was explored. Our results suggested that the improved 2SFCA methods were superior to the traditional 2SFCA method, and displayed more accessibility gradient information. We found that different groups have experienced inequity in park green space services. Among them, the inequity of youth’s enjoyment of park green space services was particularly remarkable. In addition, we have identified communities where park green space did not match population needs, such as “low population density–high accessibility” and “high population density–low accessibility”. The spatial relationship between park green space accessibility and housing prices was revealed. Our study can provide enlightenment for urban managers and planners to understand the current shortage of park green space services and where to add them. It is conducive to promoting the balanced setting of urban public services and reducing the gap between different social strata in enjoying public service resources in the post-COVID-19 Era.

## Figures and Tables

**Table 1:** Average accessibility value and the rank of park green space in various districts of Wuhan.

	Methods	2SFCA		RKD2SFCA		RG2SFCA		RA2SFCA	
Districts		Value	Rank	Value	Rank	Value	Rank	Value	Rank
Qingshan		0.093	1	0.099	2	0.089	2	0.097	2
Hanyang		0.088	2	0.102	1	0.090	1	0.098	1
Jiang’an		0.062	3	0.067	3	0.042	4	0.078	3
Wuchang		0.058	4	0.059	4	0.049	3	0.059	4
Hongshan		0.051	5	0.050	5	0.039	5	0.042	5
Qiaokou		0.039	6	0.038	6	0.028	6	0.039	6
Jianghan		0.030	7	0.032	7	0.023	7	0.031	7

**Table 2:** Summary of the associations between park green space accessibility (*A_i_*) and population density (*Pd_i_*).

		High *Pd_i_*-High *A_i_*	High *Pd_i_*-Low *A_i_*	Low *Pd_i_*-Low *A_i_*	Low *Pd_i_*-High *A_i_*
Youth	Number of communities	143	142	186	100
	Proportion of communities	12.08%	12.00%	15.71%	8.45%
	Population proportion	21.35%	29.61%	3.45%	1.92%
Young and middle-aged	Number of communities	133	136	211	100
	Proportion of communities	11.23%	11.49%	17.82%	8.45%
	Population proportion	19.36%	25.93%	4.82%	2.32%
Elderly	Number of communities	162	104	220	91
	Proportion of communities	13.68%	8.78%	18.58%	7.69%
	Population proportion	24.38%	17.19%	4.84%	1.80%

**Table 3:** Summary of the associations between park green space accessibility (*A_i_*) and housing price (*Hp_i_*).

Association	High *Hp_i_*-High *A_i_*	High *Hp_i_*-Low *A_i_*	Low *Hp_i_*-Low *A_i_*	Low *Hp_i_*-High *A_i_*
Number of communities	143	103	224	115
Proportion of communities	12.08%	8.70%	18.92%	9.71%
Population proportion	13.24%	12.65%	12.83%	8.82%

## Data Availability

Not applicable.
